# Real‐world evidence of tisagenlecleucel for the treatment of relapsed or refractory large B‐cell lymphoma

**DOI:** 10.1002/cam4.3881

**Published:** 2021-05-01

**Authors:** Gloria Iacoboni, Guillermo Villacampa, Nuria Martinez‐Cibrian, Rebeca Bailén, Lucia Lopez Corral, Jose M. Sanchez, Manuel Guerreiro, Ana Carolina Caballero, Alberto Mussetti, Juan‐Manuel Sancho, Rafael Hernani, Pau Abrisqueta, Carlos Solano, Anna Sureda, Javier Briones, Alejandro Martin Garcia‐Sancho, Mi Kwon, Juan Luis Reguera‐Ortega, Pere Barba, GETH, GELTAMO Spanish Groups

**Affiliations:** ^1^ Department of Hematology University Hospital Vall d'Hebron Barcelona Spain; ^2^ Department of Medicine Universitat Autònoma de Barcelona Bellaterra Spain; ^3^ Experimental Hematology Vall d’Hebron Institute of Oncology (VHIO) Barcelona Spain; ^4^ Oncology Data Science Vall d’Hebron Institute of Oncology (VHIO) Barcelona Spain; ^5^ Department of Hematology University Hospital Virgen del Rocio Sevilla Spain; ^6^ Department of Hematology Hospital General Universitario Gregorio Marañón Madrid Spain; ^7^ Gregorio Marañón Health Research Institute (IiSGM) Madrid Spain; ^8^ Hematology Department Hospital Clínico Universitario de Salamanca IBSAL, CIBERONC Salamanca Spain; ^9^ Centro de Investigación del Cáncer‐IBMCC Salamanca Spain; ^10^ Hematology Department Hospital 12 de Octubre Madrid Spain; ^11^ Department of Hematology Hospital La Fe Valencia Spain; ^12^ Hematology Department Hospital de la Santa Creu i Sant Pau Barcelona Spain; ^13^ Josep Carreras Leukaemia Research Institute Barcelona Spain; ^14^ Hematology Department Institut Catala d'Oncologia Hospital Duran i Reynals, L'Hospitalet De Llobregat Spain; ^15^ Institut d'Investigació Biomèdica de Bellvitge (IDIBELL) L'Hospitalet De Llobregat Barcelona Spain; ^16^ Hematology Department ICO‐IJC Hospital Germans Trias i Pujol Barcelona Spain; ^17^ Department of Hematology Hospital Clínico Universitario de Valencia Valencia Spain; ^18^ Instituto de Investigación Sanitaria INCLIVA Valencia Spain; ^19^ Department of Medicine University of Valencia Valencia Spain

**Keywords:** clinical cancer research, clinical observations, hematological cancer, non‐Hodgkin's lymphoma

## Abstract

Tisagenlecleucel (tisa‐cel) is a second‐generation autologous CD19‐targeted chimeric antigen receptor (CAR) T‐cell therapy approved for relapsed/refractory (R/R) large B‐cell lymphoma (LBCL). The approval was based on the results of phase II JULIET trial, with a best overall response rate (ORR) and complete response (CR) rate in infused patients of 52% and 40%, respectively. We report outcomes with tisa‐cel in the standard‐of‐care (SOC) setting for R/R LBCL. Data from all patients with R/R LBCL who underwent leukapheresis from December 2018 until June 2020 with the intent to receive SOC tisa‐cel were retrospectively collected at 10 Spanish institutions. Toxicities were graded according to ASTCT criteria and responses were assessed as per Lugano 2014 classification. Of 91 patients who underwent leukapheresis, 75 (82%) received tisa‐cel therapy. Grade 3 or higher cytokine release syndrome and neurotoxicity occurred in 5% and 1%, respectively; non‐relapse mortality was 4%. Among the infused patients, best ORR and CR were 60% and 32%, respectively, with a median duration of response of 8.9 months. With a median follow‐up of 14.1 months from CAR T‐cell infusion, median progression‐free survival and overall survival were 3 months and 10.7 months, respectively. At 12 months, patients in CR at first disease evaluation had a PFS of 87% and OS of 93%. Patients with an elevated lactate dehydrogenase showed a shorter PFS and OS on multivariate analysis. Treatment with tisa‐cel for patients with relapsed/refractory LBCL in a European SOC setting showed a manageable safety profile and durable complete responses.

## Graphical abstract

This article provides real‐world European data on the results of relapsed/refractory large B‐cell lymphoma patients treated with tisagenlecleucel.

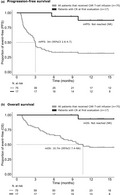

## 1 INTRODUCTION

First‐line immunochemotherapy cures around 60% of patients with large B‐cell lymphoma (LBCL).^1^, ^2^ In the relapse/refractory (R/R) setting, second‐line immunochemotherapy, usually including autologous stem cell transplant consolidation, salvages less than half of the patients,^3^, ^4^, ^5^, ^6^ whereas those in the third‐line setting have a dismal prognosis.^7^

Chimeric antigen receptor (CAR) T‐cell therapy provides long‐term remissions in a proportion of patients with R/R LBCL with significant but manageable toxicity. The results of two pivotal phase 2 clinical trials led to the approval of axicabtagene ciloleucel (axi‐cel) and tisagenlecleucel (tisa‐cel) by the Food and Drug Administration (FDA) and European Medicines Agency (EMA) for patients with R/R LBCL after 2 or more lines of systemic therapy. These trials had strict inclusion criteria and infused around 100 patients each, mainly in United States (US).^8^, ^9^, ^10^ Thus, evaluating the feasibility of this therapy outside the US, as well as gaining knowledge on the outcome of patients treated in the commercial setting is mandatory.

Several single‐center and registry‐based studies have shown that treatment with axi‐cel is feasible outside the clinical trial setting with a similar safety and efficacy profile to the pivotal trial.^11^, ^12^, ^13^, ^14^ However, data regarding the use of tisa‐cel in patients with R/R LBCL outside clinical trials are scarce.^13^, ^14^, ^15^

To provide valuable information on patient outcomes with commercial tisa‐cel in LBCL, we performed a national, multicenter, retrospective study evaluating the safety and efficacy of tisa‐cel in a European real‐life setting.

## 2 METHODS

### 2.1 Data collection and analysis

Data were collected retrospectively on all consecutive patients with R/R LBCL who underwent leukapheresis with the intent to manufacture commercial tisa‐cel at 10 Spanish institutions from December 1st, 2018, until June 1st, 2020.

For the safety analysis, we included all patients who received a tisa‐cel infusion and had a minimum follow‐up of 1 month. Efficacy‐evaluable patients included those who met the prior criteria and had an imaging response assessment. Survival outcomes were assessed in all patients who underwent leukapheresis (intention‐to‐treat analysis, ITT) and in patients who received a CAR T‐cell infusion. The study was approved by the ethics committee of the Vall d’Hebron Hospital Board.

### 2.2 Patient management

Patients were selected by hematologists around the country when they met technical data sheet criteria. A checklist with the usual screening tests (PET scan, laboratory results, echocardiogram, repeat biopsy if applicable) was forwarded to the Spanish Ministry of Health, who reviewed the proposal. Once it received approval, apheresis was performed. Bridging treatment was usually carried out at the local hospital.

Lymphodepleting (LD) chemotherapy included three consecutive days of fludarabine (25 mg/m^2^/day) and cyclophosphamide (250 mg/m^2^/day) in all cases and started once tisa‐cel had arrived on site; if the CAR T‐cell product did not meet commercial release criteria according to EMA requirements (out‐of‐specification, OOS) but it was considered acceptable by the physician, patients were offered treatment through an expanded access protocol and their results were included. After 2–4 days of chemotherapy washout, patients received the CAR T‐cell infusion in a hospitalization regimen to guarantee a close monitoring of adverse events, such as cytokine release syndrome (CRS) and immune effector cell‐associated neurotoxicity syndrome (ICANS). Management of these adverse events was carried out according to the institutional guidelines in each center. Infectious complications were managed homogenously according to the Spanish consensus guidelines.^16^

For the efficacy analysis, all patients underwent a baseline Positron Emission Tomography and Computed Tomography (PET/CT) scan immediately before the start of LD chemotherapy (after the last bridging regimen) at the infusing center. Disease evaluation after CAR T‐cell therapy was scheduled at 1, 3, 6, 12, 18, and 24 months after infusion. The imaging reports were based on the Lugano recommendation for response assessment,^17^ and PET images were graded according to the 5‐point Deauville score.

### 2.3 Definitions and endpoints

Disease status at leukapheresis was defined as one of three possibilities: (a) primary refractory if never achieving end‐of‐treatment CR; (b) refractory to last therapy if not primary refractory but not achieving a complete response to the most recent therapy, (c) or relapsed. Bridging therapy was defined as any lymphoma‐specific treatment administered after leukapheresis and before lymphodepleting chemotherapy.

Grading of CRS and ICANS was performed following the American Society for Transplantation and Cellular Therapy (ASTCT) criteria.^18^ Patients who were infused before April 2019 were graded according to the Lee criteria^19^ and then re‐assessed retrospectively to meet the 2019 criteria. Severe CRS and/or ICANS were defined as grade 3 or higher events. For the reporting of other adverse events, Common Terminology Criteria for Adverse Events (CTCAE) version 5.0 was used.^20^ Tumor lysis syndrome was defined according to Cairo‐Bishop criteria.^21^

Objective response rate (ORR) was defined as the percentage of patients who achieved a partial remission (PR) or complete remission (CR) after CAR T‐cell infusion. Progression‐free survival (PFS) was defined as the time from apheresis (ITT population) or CAR T‐cell infusion until relapse, progression, or death from any cause. Overall survival (OS) was defined as the time from apheresis (ITT) or CAR T‐cell infusion until death of any cause. The duration of response (DOR) was defined as the time from CR or PR to relapse, progression, or death from any cause, whichever occurred first.

### 2.4 Statistical analyses

A descriptive analysis of all included variables in the study was performed. Continuous variables were expressed as median and interquartile range (IQR), and categorical variables were expressed as absolute values and percentages. Univariate logistic regression model was carried out to estimate the association between ORR and baseline factors. Survival analysis (PFS and OS) was calculated using the Kaplan–Meier method and the log‐rank test was used for statistical comparison. Cox proportional hazard models were used to obtain hazard ratios (HRs) with 95% CIs. For variable selection in multivariate analysis, we used the least absolute shrinkage and selection operator (LASSO) method to construct the most parsimonious model.^22^ To assess the importance of the type of response at first evaluation, a landmark analysis using the date of first evaluation in non‐progressor patients was performed.^23^

The data analyses were carried out using R statistical software version 3.6.2.

## 3 RESULTS

### 3.1 Patients and product characteristics

Ninety‐one patients with R/R LBCL underwent leukapheresis for tisa‐cel. Seventy‐five (82%) patients received the CAR T‐cell infusion, whereas 16 (18%) did not. Reasons to not receive the infusion were: progressive disease (n = 11, 69%), manufacturing failure (n = 4, 25%), and psychiatric disorder (n = 1, 6%). All 75 patients had at least the first disease response evaluation at 1‐month post‐infusion.

Baseline characteristics of the infused patients are summarized in Table 1. The median age was 60 years (IQR 52–67) and 59% were male patients. Most had an International Prognostic Index score >2 (62%), an advanced stage (92%) and were primary refractory (52%). Sixty‐five patients (87%) received bridging therapy before infusion, including chemotherapy in most cases (n = 56, 86%). The median time from apheresis to infusion was 53 days (IQR 49–56). Median infused cell dose was 3.5 × 10^8^ CAR positive viable T‐cells (IQR 1.5–4.2). Eight products were considered OOS according to EMA requirements and four could not be manufactured. Reasons for OOS were low cellularity (n = 6, 75%) and low viability (n = 2, 25%). Six of the eight OOS products were infused (Table S1), the other two patients were not infused due to rapid disease progression. Median follow‐up from CAR T‐cell infusion was 14.1 months (95%CI 13.1–17.4).

**TABLE 1:** Baseline characteristics of infused patients

Baseline characteristics of infused patients^a^	N = 75
Median age (IQR) ‐years	60 (52–67)
Age ≥65 y– n (%)	23 (31)
Gender –n (%)	
Male	44 (59)
Female	31 (41)
ECOG score, median (IQR)	1 (0–1)
0–n (%)	25 (33)
1–n (%)	41 (55)
2–n (%)	5 (7)
Missing data –n (%)	4 (5)
Histology – n (%)	
Diffuse large B‐cell lymphoma, NOS	44 (58)
High grade B‐cell lymphoma DH/TH	11 (15)
Transformed from follicular lymphoma	17 (23)
Transformed from other indolent histology	3 (4)
Cell of origin – n (%)	
GCB	44 (59)
Non‐GCB	24 (32)
Missing data	7 (9)
Disease stage‐ n (%)	
Stage I‐II	6 (8)
Stage III‐IV	69 (92)
Extranodal disease (≥1 site)–n (%)	60 (80)
Bulky disease (>7 cm)–n (%)	30 (42)
LDH levels before treatment	
<2xULN	51 (68)
≥2xULN	24 (32)
IPI prognostic score – n (%)	
0–2	25 (33)
3–5	46 (62)
Missing data	4 (5)
Number of previous lines of treatment, median (IQR)	3 (2–4)
2–3	54 (72)
>3	21 (28)
Previous ASCT – n (%)	29 (39)
Response to previous therapy – n (%)	
Primary refractory	39 (52)
Refractory to last therapy	22 (29)
Relapsed	14 (19)
Bridging treatment ‐n (%)	65 (87)
Cyclophosphamide‐Prednisone/CVP^b^	29 (44)
Platinum‐based^c^	16 (24)
Bendamustine‐based^d^	5 (8)
Rituximab‐CHOP^e^	3 (5)
Steroids	2 (3)
Radiotherapy	2 (3)
Rituximab‐Lenalidomide	2 (3)
Other chemotherapy^f^	3 (5)
Data not available	3 (5)

Abbreviations: ASCT, Autologous Stem Cell Transplant; DH/TH, Double Hit/Triple Hit; ECOG, Eastern Cooperative Oncology Group; GCB, Germinal Center B‐cell; IPI, International Prognostic Index; IQR, Interquartile range; LDH, Lactate Dehydrogenase; NOS, Not Otherwise Specified; ULN, Upper Limit of Normal.

^a^ ECOG score was missing in four patients, IPI missing in four patients; Bulky data missing in two patients, extranodal missing in one patient.

^b^ CVP, Cyclophosphamide, vincristine, and prednisolone.

^c^ Platinum‐based strategies included R‐GEMOX (12), R‐GDP (3) and R‐ESHAP (1).

^d^ Bendamustine based included Rituximab‐Bendamustine with (2) or without (3) Polatuzumab.

^e^ CHOP, Cyclophosphamide, Doxorubicin, Vincristine, Prednisone.

^f^ Other chemotherapy included MINE (Mesna, Ifosfamide, Mitoxantrone, Etoposide), R‐IE (rituximab, ifosfamide, and etoposide) and R‐hyperCVAD (Cyclophosphamide, Vincristine, Adriamycin, and Dexamethasone).

### 3.2 Safety analysis

Among the infused patients, 53 (71%) developed any grade of CRS; 21 (28%) and four (5%) patients developed grade ≥2 and grade ≥3 CRS, respectively. Eleven (15%) patients developed any grade of ICANS, whereas five (7%) and one (1%) developed grade ≥2 and grade ≥3 ICANS, respectively. The median time from infusion to the onset of symptoms of CRS and ICANS was 2 days (IQR 1–4) and 7 days (IQR 5–9), respectively. Tocilizumab and steroids were administered to 24 (32%) and 16 (21%) patients, respectively. Ten (13%) patients required admission to the Intensive Care Unit. By day 90 post‐infusion, three (4%) patients experienced treatment‐related mortality: two from bacterial infection, specifically one case of Klebsiella pneumoniae BLEE sepsis and another of multiresistant Pseudomonas aeruginosa soft tissue infection (at 26 and 30 days from infusion, respectively) and one from macrophage activation syndrome (MAS)^24^ (at 36 days from infusion), despite treatment with tocilizumab, steroids, anakinra, and siltuximab. There were two other cases of MAS: one resolved with dexamethasone and the other without specific treatment. Other adverse events including infection and tumor lysis syndrome are summarized in Table 2.

**TABLE 2:** Safety analysis of infused patients

Safety profile of infused patients	N = 75
CRS	
Any grade; n (%)	53 (71)
Grade ≥2; n (%)	21 (28)
Grade ≥3; n (%)	4 (5)
Time from infusion to start of CRS; median days (IQR)	2 (1–4)
Duration CRS; median days (IQR)	4 (4–6)
ICANS	
Any grade; n (%)	11 (15)
Grade ≥2; n (%)	6 (8)
Grade ≥3; n (%)	1 (1)
Time from infusion to start of ICANS; median days (IQR)	7 (5–9)
Duration ICANS; median days (IQR)	9 (3–14)
ICU, n (%)	10 (13)
Tocilizumab	
Patients; n (%)	24 (32)
Median number doses tocilizumab (IQR)	1 (1–5)
Corticosteroids	
Patients; n (%)	16 (21)
Duration steroids; median days (IQR)	11 (8–15)
Macrophage activation syndrome, n (%)	3 (4)
Infections in the first month after infusion	
Patients, n (%)	21 (28)
Infectious events	31
Bacterial	19
Viral	8
Fungal	3
Not identified	1
Tumor Lysis Syndrome, n (%)	2 (3)
Treatment‐related mortality, n (%)	3 (4)

Abbreviations: Cytokine Release Syndrome (CRS); Immune Effector Associated Neurotoxicity Syndrome (ICANS); Intensive Care Unit (ICU); Interquartile range (IQR).

The univariate analysis of risk factors for the development of adverse events is summarized in Table S2. The baseline characteristics associated with an increased risk of grade ≥2 CRS and/or ICANS were ECOG (≥1 vs. 0), primary refractory disease, lactate dehydrogenase (LDH) levels (>2xULN [(Upper Limit of Normal)] vs. <2xULN), and the infused cell dose per kg of body weight (0.01‐units increase).

### 3.3 Efficacy analysis

#### 3.3.1 Disease response

Among the 75 infused patients, the best response achieved was CR in 24 (32%) patients and PR in 21 (28%), with an ORR of 60%. In the ITT analysis, the best response achieved was CR in 26% (24/91) with an ORR of 49% (45/91). Patients who achieved a response (CR or PR) had a median duration of response of 8.9 months (95%CI 2.2–NA). Stable disease and progressive disease were the best response in 6 (8%) and 24 (32%) patients, respectively. Of the six infused OOS products, two patients achieved a CR, one patient achieved a stable disease and eventually progressed, whereas three patients progressed at the first disease assessment (Table S1).

Regarding the patients who achieved an initial PR at the 1‐month disease assessment, five (5/25, 20%) converted to CR at 3 (2 patients), 6, 12, and 18 months, respectively; the other patients in PR progressed in the following 3 to 6 months (18/25, 72%), or were in a maintained PR at data cutoff (2/25, 8%). Regarding the nine patients who achieved an initial SD, two patients converted to a CR at 6‐ and 18‐months post‐infusion, respectively, and one patient improved to a PR at 3 months post‐infusion; one patient remained in SD at 6 months post‐infusion and the remaining five patients had progressed at data cutoff.

**FIGURE 1:**
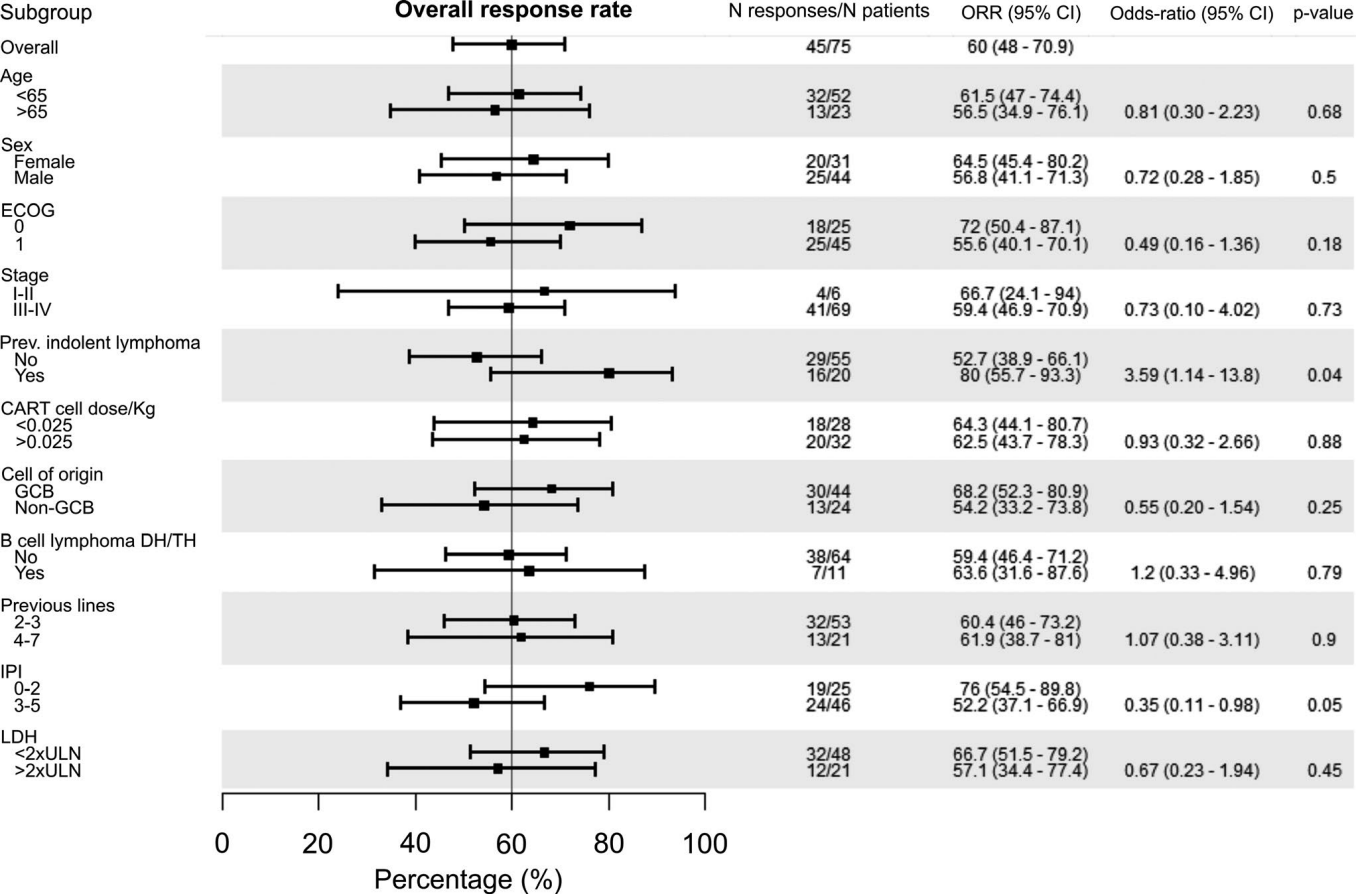
Subgroup analysis according to ORR for infused patients. Note: Overall response rate according to baseline patient and disease characteristics

In the subgroup analysis, ORR was consistent across all baseline characteristics except for the International Prognostic Index (IPI) score (a high IPI [3–5] showed a lower ORR [OR 0.35, *p *= 0.05]) and a history of previous indolent lymphoma (OR 3.59, *p *= 0.04) (Figure 1). In the subgroup analysis for CR, patients with an ECOG of 0, an LDH <2xULN and a low IPI score had a significantly increased probability of achieving a CR (Figure S1). There was no significant difference in any of the efficacy endpoints for patients with high‐grade B‐cell lymphoma (HGBL) with *MYC* and *BCL2* and/or *BCL6* rearrangements.

Patients who developed grade 2 or higher CRS or ICANS had a similar ORR (65% vs. 58%, *p *= 0.54) and OS in comparison to patients who did not develop grade 2 or higher adverse events. There was no significant impact of tocilizumab or steroid use on the ORR (69% and 81%, respectively, *p*‐values >0.1).

#### 3.3.2 Survival analysis

**FIGURE 2:**
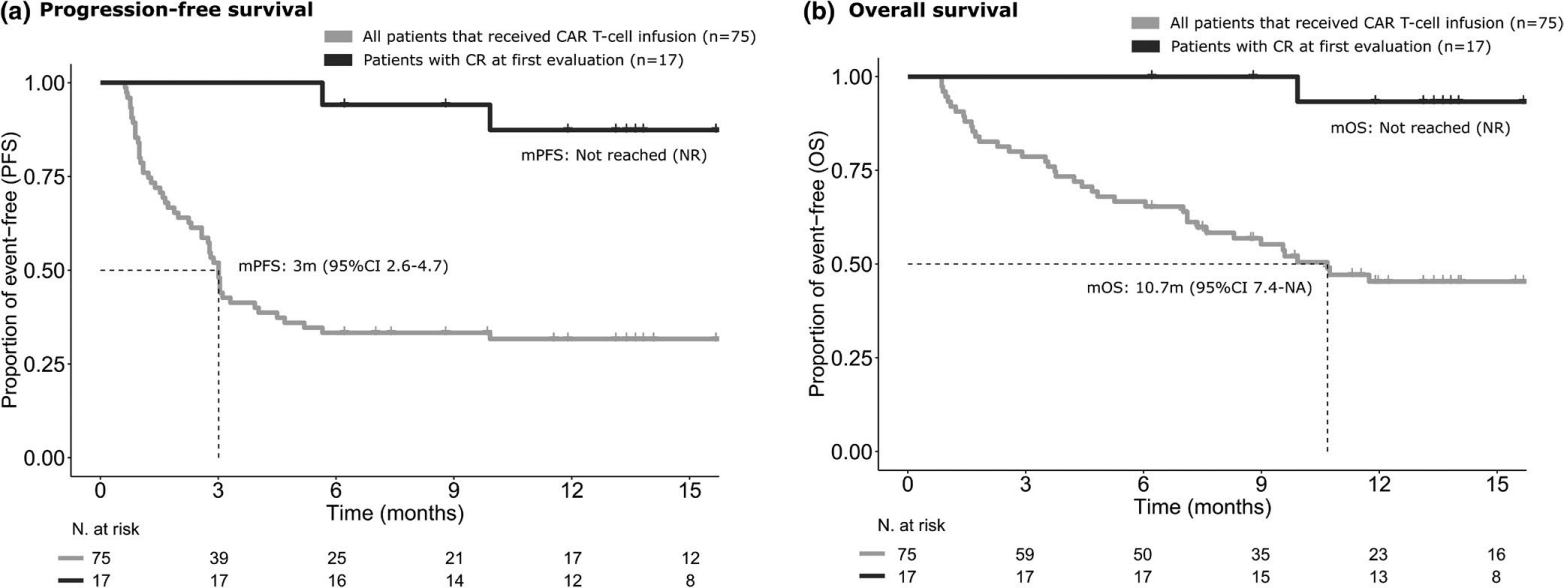
PFS (a) and OS (b) for all infused patients and for patients achieving CR at first disease assessment. Note: PFS and OS in infused patients and in patients achieving CR after CAR T‐cell infusion

Median PFS and OS for all infused patients were 3 months (95%CI 2.6–4.7) and 10.7 months (95%CI 7.4–NA), respectively (Figure 2). The overall 6‐month and 12‐month PFS was 33.3% and 31.7%, respectively. Patients in CR and PR at first disease evaluation had a PFS at 12 months of 87% and 20% (*p *< 0.001) and OS of 93% and 39% (*p *< 0.001), respectively (Figure S2).

In the ITT analysis, median PFS and OS from apheresis were 4.6 months (95%CI 4.1–6.9) and 11.1 months (95%CI 7.9–NA), respectively (Figure S3).

**FIGURE 3:**
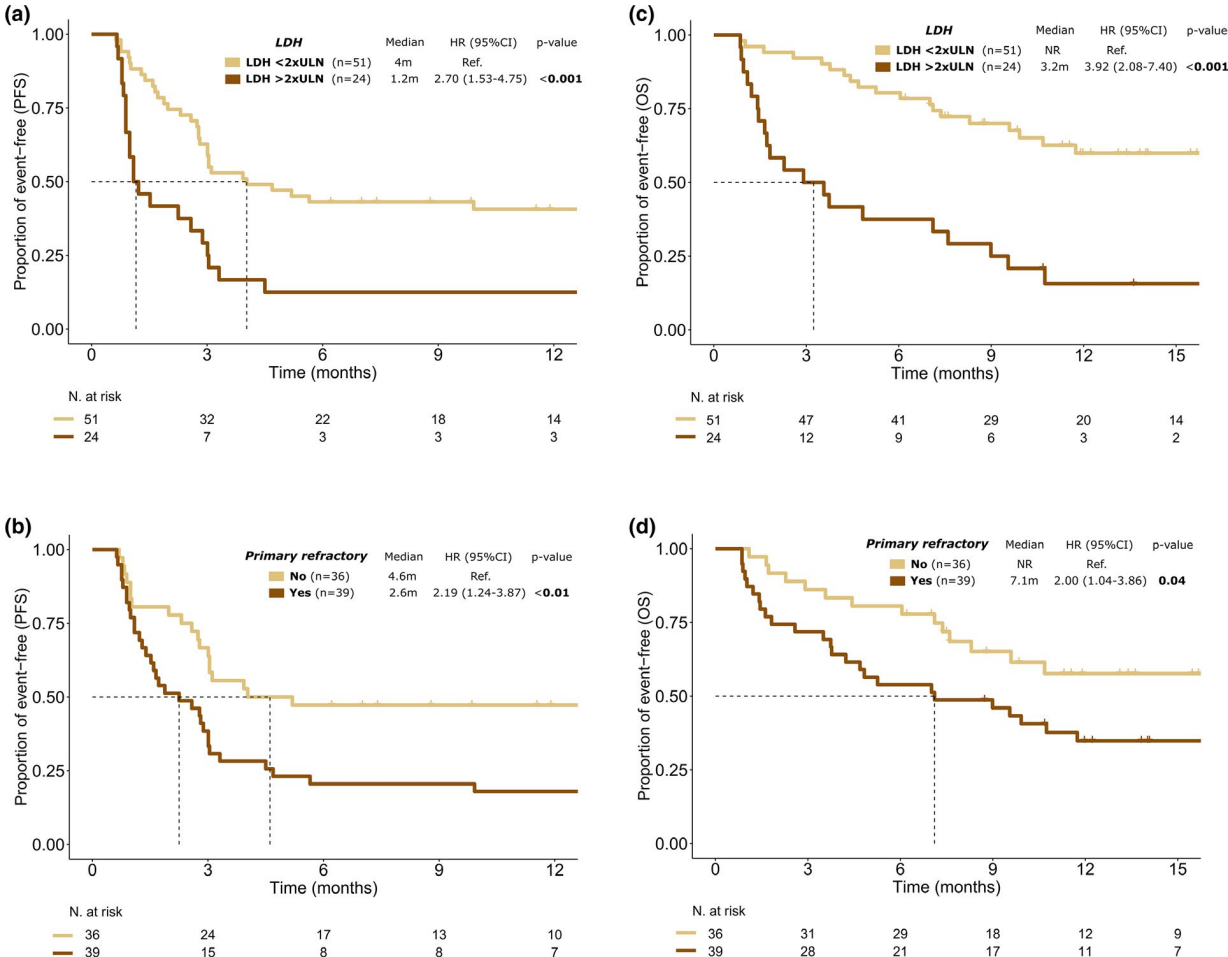
Impact of LDH levels and primary refractory disease on PFS and OS of infused patients. Note: Impact of LDH levels on PFS (a) and OS (c); impact of primary refractory disease on PFS (b) and OS (d)

In terms of PFS, patients who were primary refractory, had an ECOG of 1 or higher, a non‐GCB cell of origin, high IPI score and high LDH levels (>2xULN) had a significantly lower PFS in the univariate analysis (Table 3 and Figure 3). Primary refractory disease [HR: 2.24 (95%CI 1.20–4.18), *p *= 0.01] and high LDH levels [HR: 2.18 (95%CI 1.19–3.99), *p *= 0.01] maintained the independent statistical significance in the multivariate model (Table 3).

**TABLE 3:** Univariate and multivariate analysis of risk factors for PFS and OS

	Progression‐free survival (n = 75, events = 51)				Overall survival (n = 75, events = 39)			
	Univariate analysis		Multivariate analysis		Univariate analysis		Multivariate analysis	
	HR (95% CI)	*p* value	HR (95% CI)	*p* value	HR (95% CI)	*p* value	HR (95% CI)	*p* value
**Age** (10‐years increase)	0.82 (0.65–1.04)	0.10	—	—	0.78 (0.59–1.02)	0.07	—	—
**Sex** (male vs. female)	1.30 (0.73–2.29)	0.37	—	—	1.37 (0.71–2.64)	0.34	—	—
**ECOG** (1+ vs. 0)	1.97 (1.06–3.68)	**0.03**	—	—	4.23 (1.75–10.2)	**<0.01**	2.83 (1.12–7.11)	**0.03**
**Stage** (III‐IV vs. I‐II)	1.54 (0.55–4.28)	0.41	—	—	2.24 (0.54–9.29)	0.27	—	—
**Prev. indolent lymphoma** (yes vs. no)	0.89 (0.47–1.67)	0.71	—	—	0.90 (0.44–1.85)	0.78	—	—
**Primary refractory** (yes vs. no)	2.19 (1.24–3.87)	**<0.01**	1.99 (1.12–3.55)	**0.02**	2.00 (1.04–3.86)	**0.04**	1.79 (0.90–3.56)	0.09
**Bulky** (>7 cm vs. <7 cm)	1.56 (0.88–2.75)	0.13	—	—	1.86 (0.96–3.58)	0.06	—	—
**Cell of origin** (Non‐GCB vs. GCB)	1.70 (0.93–3.09)	0.08	—	—	1.81 (0.92–3.57)	0.09	—	—
**HGBL** (yes vs. no)	0.99 (0.45–2.19)	0.98	—	—	1.31 (0.58–2.96)	0.52	—	—
**Previous lines**	1.05 (0.83–1.33)	0.69	—	—	1.17 (0.88–1.54)	0.28	—	—
**IPI score**	1.74 (1.30–2.31)	**<0.001**	*	*	1.79 (1.28–2.51)	**<0.001**	*	*
**CAR T‐cell dose**	0.81 (0.58–1.12)	0.21	—	—	0.69 (0.46–1.02)	0.07	—	—
**CAR T‐cell dose/ kg** (0.01‐units increase)	0.86 (0.68–1.07)	0.18	—	—	0.81 (0.62–1.06)	0.12	—	—
**LDH** (>2xULN vs. <2xULN)	2.70 (1.53–4.86)	**<0.001**	2.48 (1.40–4.40)	**<0.01**	3.92 (2.08–7.40)	**<0.001**	2.75 (1.38–5.46)	**<0.01**

*IPI score was not included in the multivariate analysis because of the high multicollinearity with other covariates. IPI score and CAR T‐cell dose were analyzed as continuous variables.

Abbreviations: CART, Chimeric Antigen Receptor T‐cell; ECOG, Eastern Cooperative Oncology Group; GCB, Germinal Center B‐cell; HGBL, High‐grade B‐cell lymphoma; IPI, International Prognostic Index; LDH, Lactate Dehydrogenase; ULN, Upper Limit of Normal.

For OS, patients who were primary refractory, had an ECOG of 1 or higher, high IPI score and high LDH levels were associated with a lower OS in the univariate analysis (Table 3 and Figure 3). In the multivariate analysis, ECOG [HR: 2.80 (95%CI 1.10–7.11), *p *= 0.03] and LDH levels [HR: 2.33 (95%CI 1.14–4.76), *p *= 0.02] remained significantly associated with OS (Table 3).

## 4 DISCUSSION

This is the largest study to date focused exclusively on patients with R/R LBCL treated with tisa‐cel in the real‐world setting. We have shown that CAR T‐cell therapy with tisa‐cel is feasible outside the US and has a similar safety and efficacy profile to the pivotal clinical trial.^9^

In our study, 91 patients with R/R LBCL underwent leukapheresis for commercial tisa‐cel and 75 (82%) patients received an infusion. The median time from apheresis to infusion was similar to the registration trial^9^ (54 days vs. 53 days) and in both studies most patients received bridging therapy (92% vs. 87%). This turnaround time is longer than the published real‐world data with axi‐cel and tisa‐cel in the US^11^, ^12^, ^15^ but similar to previous tisa‐cel reports from European centers.^13^, ^14^ Reasons behind this delay would include limited referral experience, reduced number of manufacturing slots, and few European facilities; this could have certainly played a role in the number of patients who dropped out due to disease progression and, even, in the final outcome of the infused patients. This bridging period has gradually improved with the increasing experience of referral sites and a growing number of European manufacturing facilities.

Eight products were considered OOS in our study, mainly due to low cellularity. Low viability, a larger problem in the US, was not as frequent, probably due to the lower EMA limit in comparison with the FDA (70% vs. 80% respectively).^15^ In our study, 82% of the patients who underwent apheresis received an infusion; in the pivotal trial^9^ only 67% of enrolled patients received an infusion, possibly related to the stricter criteria and the different baseline characteristics, which included a smaller number of transformed patients in comparison to our cohort (19% vs. 27%, respectively).

Focusing on the safety analysis, the incidence of severe adverse events, including both CRS (5%) and ICANS (1%), was lower than the JULIET trial (22% and 12%, respectively), taking into account the usual caveats derived from different grading systems.^25^, ^26^ Noteworthy, our results were similar to a recent real‐world registry‐based study including patients with LBCL and acute lymphoblastic leukemia treated with tisa‐cel in the US.^15^ Reasons behind this trend for a better safety profile in the standard‐of‐care setting compared with the clinical trial includes an increased use of tocilizumab and steroids^13^, ^15^ and more experience in CAR T‐cell management.^27^ Preliminary data from European centers seem to confirm this lower rate of severe adverse events in the commercial setting.^13^, ^28^ The median time to onset of CRS and ICANS was similar between our study and the registration trial (2 vs. 3 days and 7 vs. 6 days, respectively). Patients with primary refractory disease, a higher infused cell dose *per* kg of body weight, an ECOG ≥1 and elevated LDH levels showed an increased risk of developing grade 2 or higher adverse events. In line with the pivotal CAR T‐cell trials,^8^, ^9^ older age was not associated with an increased risk of grade ≥2 toxicity, suggesting this is a feasible treatment modality for elderly patients with R/R LBCL. There was a 4% of treatment‐related mortality in this study; however, all three patients were in progressive disease at the time of these events so this could have played a role in the final outcome.

Efficacy results in our study were similar to the pivotal trial. Interestingly, 12‐month PFS for patients who achieved a CR at first disease assessment was 87%, confirming that most patients in this subgroup will maintain their response over time. However, the PR to CR conversion rate was lower in our study than in the pivotal trial^9^ (20% vs. 54%). We also observed that patients with a SD or PD as the best response after CAR T‐cell therapy represent a high‐risk subgroup: the 12‐month OS was 14% for patients with PD/SD, in comparison to 28% for patients with PR and 95% for patients with CR. Therefore, patients in CR after tisa‐cel therapy seem to have a very good prognosis, with durable remissions in most cases; patients who achieve a PR should be closely monitored during the first 3 months, the highest‐risk period, to look out for early signs of progression and start additional therapies as soon as needed.

Patients with high LDH levels had a worse outcome after treatment with tisa‐cel. Other studies also identified LDH^11^ and high tumor volume, measured on CT^29^ or PET scan,^14^, ^30^ as clear prognostic factors for disease response after CAR T‐cell therapy. Patients with primary refractory disease also had a lower PFS and OS. In line with previous publications, we found no significant efficacy difference for HGBL patients. Taking all this into account, patients progressing after second‐line therapy with low tumor burden, a good performance status, and low LDH could potentially benefit most from this treatment.

There are some limitations to this study. The data were collected retrospectively and many of the baseline characteristics were captured before apheresis. Also, disease evaluation was only available previous to lymphodepleting chemotherapy; thus, it was not possible to assess the impact of disease response to bridging treatment as a prognostic factor for the efficacy of CAR T‐cell therapy. Longer follow‐up is needed to confirm the long‐term duration of complete remissions and safety after tisa‐cel therapy.

To the best of our knowledge, this is the largest standard‐of‐care cohort of patients reported to date with tisa‐cel for R/R LBCL in a European country. Our results confirm that treatment with tisa‐cel in Europe is feasible and has similar results to the pivotal trial.

## COMPLIANCE WITH ETHICAL STANDARDS

Ethical approval: All procedures performed in this study involving human participants were in accordance with the ethical standards of the institutional research committee and with the 1964 Helsinki declaration and its later amendments or comparable ethical standards.

Ethical approval was granted by the Vall d’Hebron Hospital Ethical Committee, study identified with code PR(AG)404/2020.

## CONFLICTS OF INTEREST

G.I. declares having received honoraria from BMS/Celgene, Gilead, Novartis, Janssen, and Roche, not related with this article. G.V. reported receiving honoraria for speaker activities from Merck Sharp & Dohme and an advisory role from Astrazeneca. A.M. declares having received Gilead research fundings and honoraria from Novartis and Takeda. JM. S. declares having received honoraria from Roche, Novartis, Gilead, Celgene, Janssen, Takeda, and Incyte; also consulting for Roche, Novartis, Gilead, Celgene, Janssen, Incyte, Celltrion, and Sandoz. P.A. has received honorarium for advisory and speaker faculty from Janssen, Roche, Celgene, Abbvie, Gilead, and Astrazeneca. A.M.G‐S. declares having received honoraria from Roche, Celgene, Janssen, Servier, Gilead; consulting fees from Roche, Celgene/BMS, Morphosys, Kyowa Kirin, Clinigen, Eusa Pharma, Novartis, Gilead, Servier; research funding from Janssen. JL. R‐O. declares having received honoraria from Novartis, Kite/Gilead, BMS/Celgene. P.B. declares having received honoraria from Amgen, Celgene, Gilead, Incyte, Jazz Pharmaceuticals, MSD, Novartis, Pfizer, and Roche, not related with this article. P.B. received funding from the Carlos III FIS16/01433 Health Institute, Asociación Española contra el Cáncer (Ideas Semilla 2019) and a PERIS 2018–2020 grant from the Generalitat de Catalunya (BDNS357800), not related to this study.

## AUTHORS’ CONTRIBUTIONS

Concept and design were undertaken by PB and GI. Data analysis and interpretation were performed by GV, PB, and GI. Collection and assembly of data were performed by all authors. All authors contributed to manuscript writing and final approval of the manuscript, and are accountable for all aspects of the work (ensuring questions related to accuracy or integrity of the work are appropriately investigated and resolved.

## Supporting information

Supplementary Material

## Data Availability

Data available on request from the authors.
