# Supplementary material for: Real‐world evidence of tisagenlecleucel for the treatment of relapsed or refractory large B‐cell lymphoma
Source: Cancer Med. 2021 May 1;10(10):3214–23. doi: 10.1002/cam4.3881 (PMC8124109; doi:10.1002/cam4.3881)
Supplement: Supplementary file 1 — Supplementary Material [file CAM4-10-3214-s001.docx]

**Supplemental material**

**Table S1.** Description of infused OOS products and patient outcome.

**Table S2.** Analysis of risk factors for grade ≥2 adverse events.

**Figure S1.** Subgroup analysis according to CR for infused patients.

Footnote Figure S1: Impact of baseline patient- and lymphoma-related characteristics on complete response rate.

**Figure S2.** PFS (A) and OS (B) for infused patients achieving CR or PR at first disease assessment.

Footnote Figure S3: Impact of achieving a CR or a PR at 1-month post-infusion on PFS (A) and OS (B).

**Figure S3**. PFS (A) and OS (B) for all patients who underwent leukapheresis.

Footnote Figure S2: PFS (A) and OS (B) for all patients who underwent leukapheresis (intention-to-treat analysis).

**Supplementary Table 1. Description of infused OOS products and patient outcome.**

| **UPN** | **Reason for OOS** | **Value** | **Outcome** |
| --- | --- | --- | --- |
| 8 | Low cellularity | 0.5 x10^8^ CAR+ viable T-cells | Complete Response |
| 15 | Low cellularity | 0.4 x10^8^ CAR+ viable T-cells | Complete Response |
| 17 | Low cellularity | 0.4 x10^8^ CAR+ viable T-cells | Progressive disease |
| 29 | Low cellularity | 0.4 x10^8^ CAR+ viable T-cells | Progressive disease |
| 18 | Low viability | 69.4% | Progressive disease |
| 74 | Low viability | 64.8% | Progressive disease |

Abbreviations: UPN, Unique Patient Number, OOS, out-of-specification.

**Supplementary Table 2. Analysis of risk factors for grade ≥2 adverse events.**

|  | **Grade ≥ 2 adverse events** | |
| --- | --- | --- |
|  | OR (95% CI) | P value |
| **Age** (10-years increase) | 0.75 (0.49 – 1.12) | 0.16 |
| **Sex** (male vs female) | 0.88 (0.33 – 2.41) | 0.80 |
| **ECOG** (1+ vs. 0) | 5.36 (1.57 – 24.9) | **0.01** |
| **Stage** (III-IV vs I-II) | 2.34 (0.35 – 46.3) | 0.45 |
| **Prev. indolent lymphoma** (yes vs. no) | 1.35 (0.43 – 4.04) | 0.59 |
| **Primary refractory** (yes vs. no) | 2.88 (1.05 – 8.61) | **0.04** |
| **Bulky** (>7cm vs <7cm) | 0.96 (0.34 – 2.64) | 0.93 |
| **Cell of origin** (Non-GCB vs. GCB) | 0.88 (0.29 – 2.57) | 0.82 |
| **Previous lines** | 0.75 (0.44 – 1.18) | 0.24 |
| **IPI score** | 1.16 (0.73 – 1.88) | 0.52 |
| **CART cell dose** | 1.26 (0.71 – 2.32) | 0.43 |
| **CART cell dose/ Kg** (0.01-units increase) | 1.48 (1.03 – 2.2) | **0.04** |
| **LDH** (>2xULN vs 2x<ULN) | 5.78 (1.92 – 18.6) | **<0.01** |

Abbreviations: Eastern Cooperative Oncology Group (ECOG); Germinal Center B-cell (GCB); International Prognostic Index (IPI); Chimeric Antigen Receptor T-cell (CART); Lactate dehydrogenase (LDH); Upper Limit of Normal (ULN)

**Supplementary Figure 1. Subgroup analysis according to CR for infused patients.**


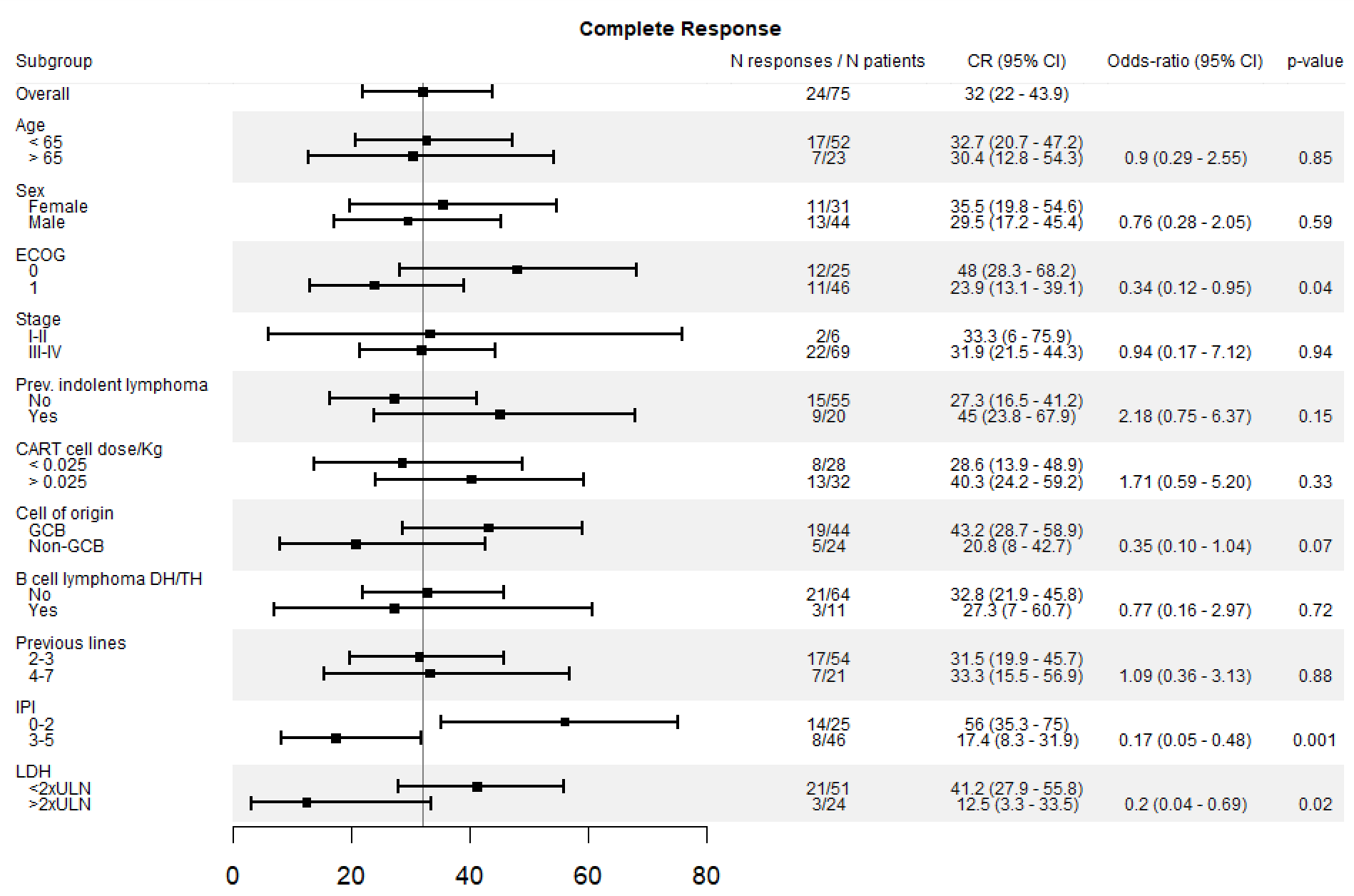


**Supplementary Figure 2. PFS (A) and OS (B) for infused patients achieving CR or PR at first disease assessment.**


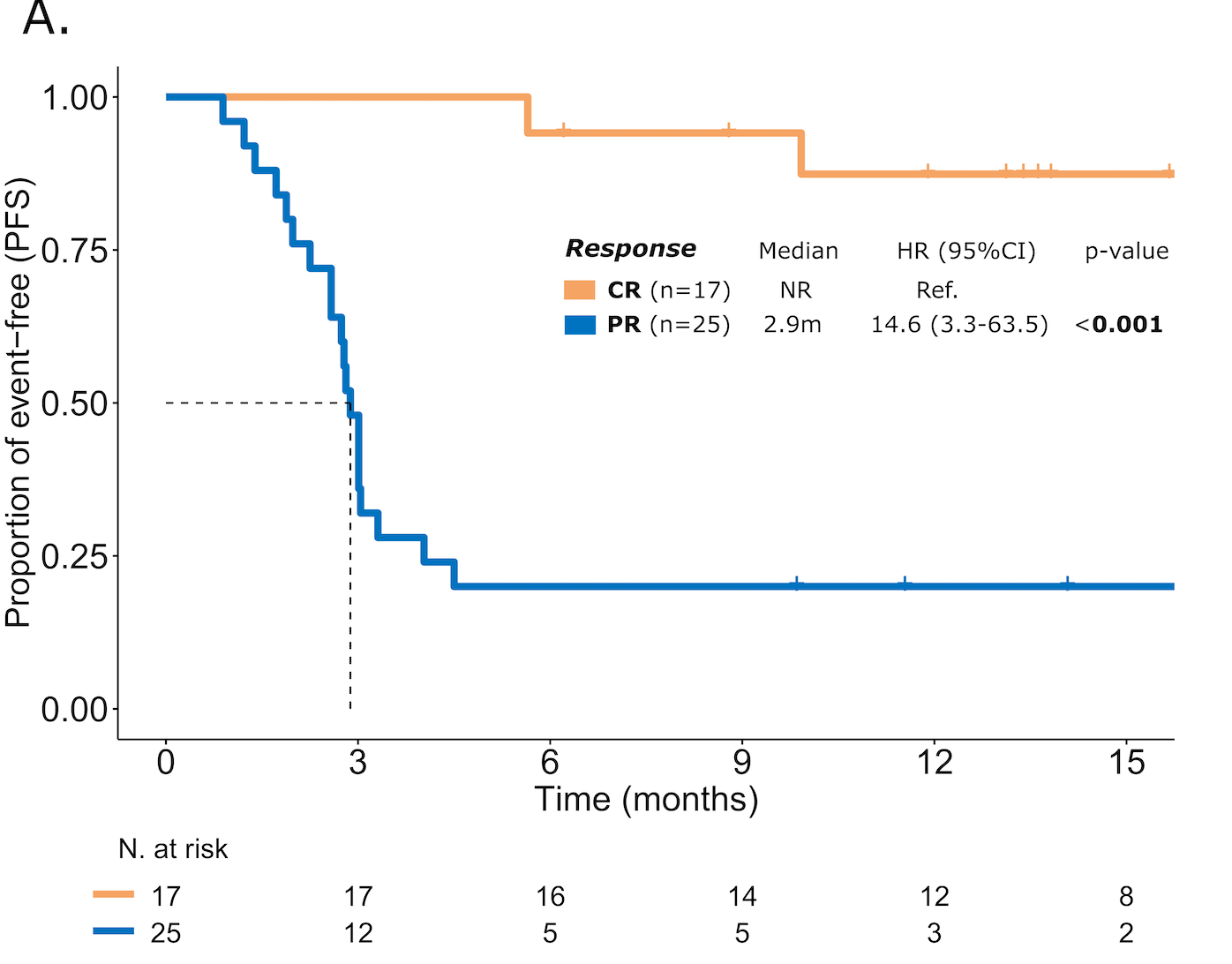


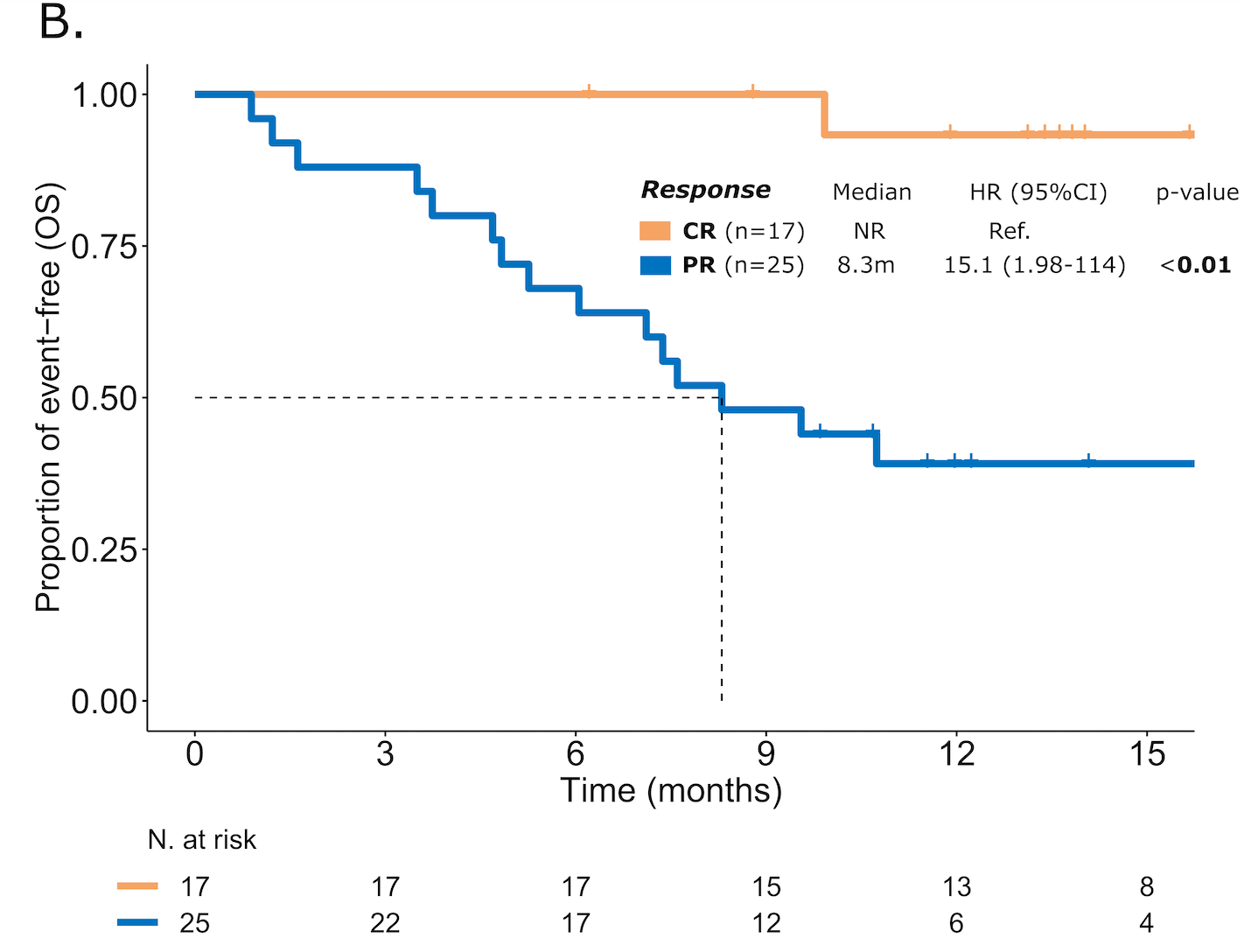


**Supplementary Figure 3. PFS (A) and OS (B) for all patients who underwent leukapheresis.**

**
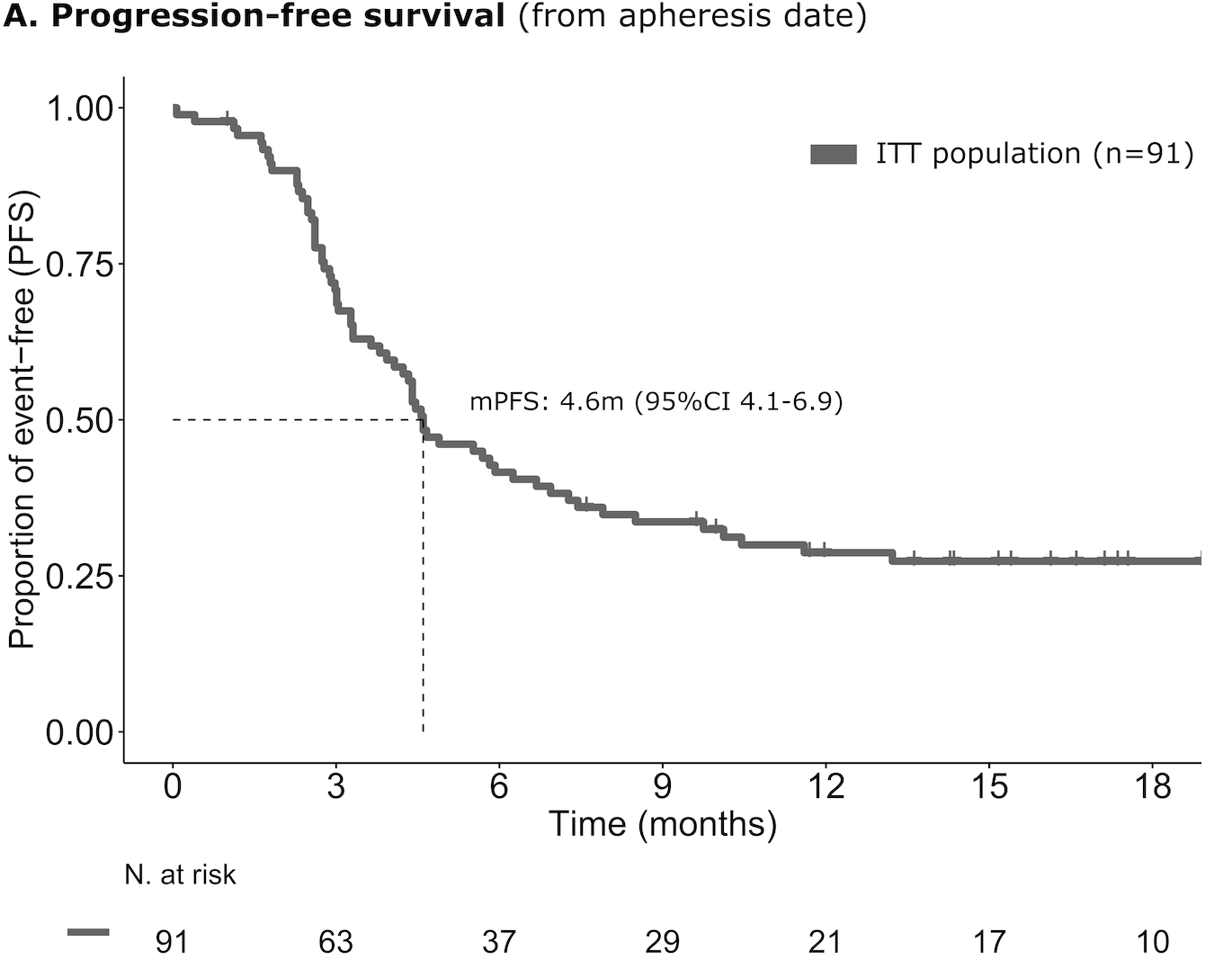
**

**
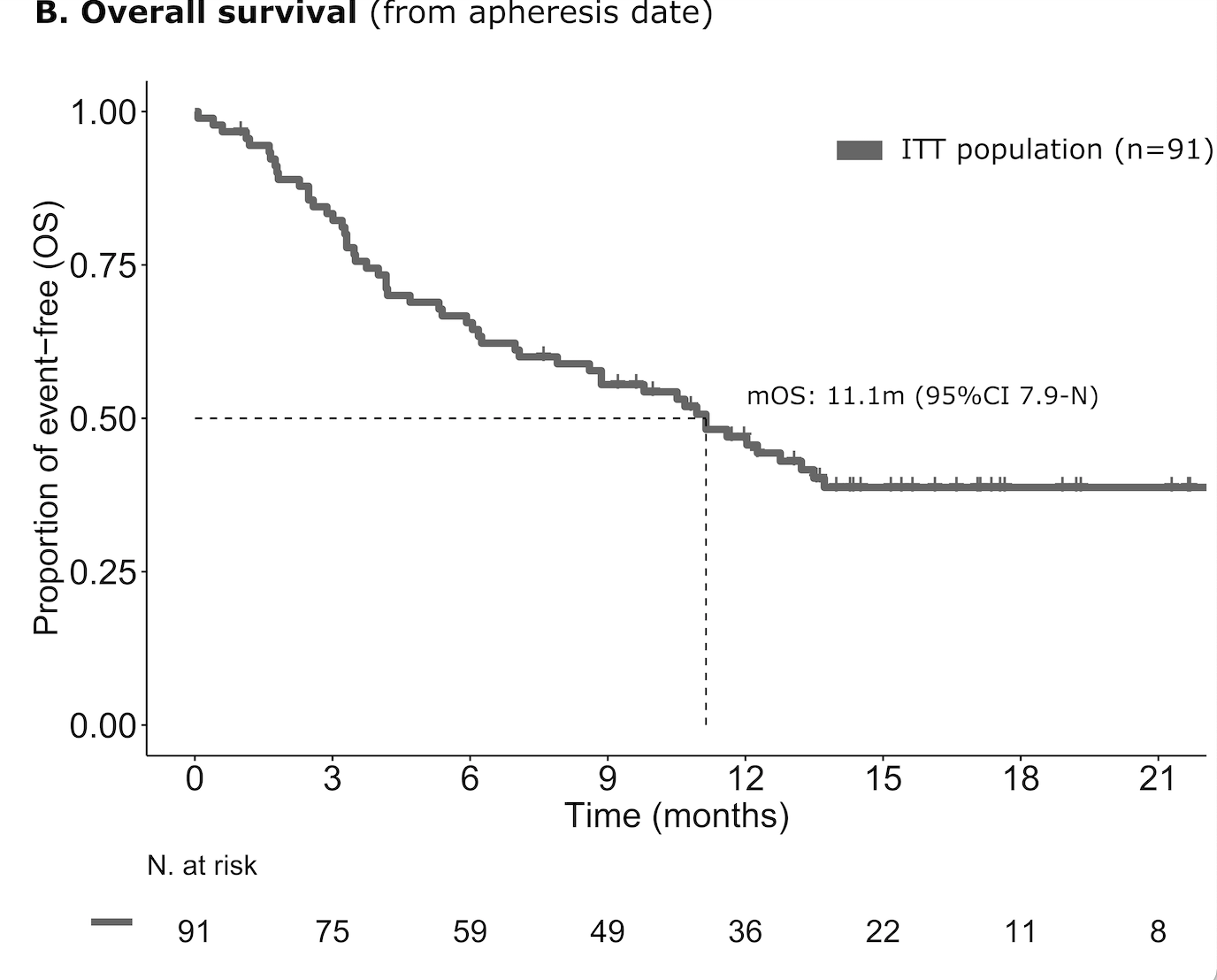
**
